# Seasonal Spatial Distribution and Migration Patterns of the Shrimp *Parapenaeus fissuroides* in the Southern Yellow and East China Seas: Habitat Area Change Under Climate Scenarios

**DOI:** 10.3390/ani15243597

**Published:** 2025-12-15

**Authors:** Min Xu, Yong Liu, Yang Xu, Haisu Zheng, Jianzhong Ling, Huiyu Li

**Affiliations:** 1 Key Laboratory of Fisheries Remote Sensing Ministry of Agriculture and Rural Affairs, East China Sea Fisheries Research Institute, Chinese Academy of Fishery Sciences, Shanghai 200090, China; xuminwzy@aliyun.com (M.X.); liuy@ecsf.ac.cn (Y.L.); yolanda202510@126.com (Y.X.); 2 Shanghai Aquatic Wildlife Conservation and Research Center, Shanghai 200080, China; zhenghaisu322@hotmail.com

**Keywords:** benthic shrimp, East China Sea, marine policy, nursery, Shared Socioeconomic Pathways, spawning, stock assessment, total allowable catch

## Abstract

Shrimp populations are threatened by overfishing and climate change. Despite this, there are limited studies on the variations in biomass, number, and size of Eastern Neptune rose shrimp, *Parapenaeus fissuroides*, in different fishing grounds across seasons in the Southern Yellow and East China Seas of China, as well as habitat area change under different climate scenarios. In this study, the data was obtained from the bottom trawling surveys conducted from November 2018 to September 2019 at 26.5–35° N 120–127° E. We found that the major cohorts were concentrated at depths of 70–110 m in spring and summer, 80–90 m in autumn, and 60–100 m in winter. The greatest abundance was observed at sea bottom temperatures of 18–20 °C in spring, 18–21 °C in summer, 19–22 °C in autumn, and 17–19 °C in winter; and sea bottom salinity levels of 34–35 across all four seasons, indicating the influence of high-salinity Taiwan Warm Current and Yellow Sea Warm Current. The annual mean catch per unit effort in weight and number were 16,623.65 g·h^−1^ and 4304.525 ind·h^−1^, respectively. Climate variations may negatively impact the habitat area range of *P. fissuroides*. For migration route, *P. fissuroides* concentrates in Yushan and Yuwai for the upcoming spawning season in spring, with offspring occurring in high-temperature and high-salinity waters of Zhouwai and Yuwai in summer. The newborn recruited cohorts remained in Yushan, Wentai, and Mindong for nursery grounds in autumn and winter. We suggested a seasonal closure from August to November in Yushan, Yuwai, Zhouwai, and Mindong to protect and conserve *P. fissuroides* populations.

## Simple Summary

Since the 1980s, the regional shrimp species *Parapenaeus fissuroides* has been exploited in the Zhejiang and Fujian regions of China. However, very little ecological information is known about the current seasonal spatial distribution patterns, as related to environmental variables, of *P. fissuroides* in the Yellow and East China Seas. In this study, independent research vessels were used to collect *P. fissuroides* biomass and abundance data across different seasons, from 2018 to 2019, covering 26.50–35.00° N and 120.00–127.00° E. We used species distribution models to predict *P. fissuroides* habitat area changes under different climate scenarios (high-emission SSP585, medium–high-emission SSP370, intermediate-emission SSP245, and low-emission SSP126) in 2040–2050 (the 2040s) and 2090–2100 (the 2090s). Our results can enhance understanding of *P. fissuroides* ecology and promote protection and conservation efforts.

## 1. Introduction

Species of the genus *Parapenaeus* (Smith 1885) are commonly distributed in tropical regions of the Atlantic and the Indo-West Pacific, usually occurring at a depth of >100 m [1]. Fifteen species and three subspecies have been reported in this genus [2]. Several species are of great economic importance and are caught commercially in the seas around China, Japan, and Korea [3]. The medium-sized species *Parapenaeus fissuroides* (Crosnier 1985) (Decapoda: Penaeidae), commonly known as ‘Jianxia,” has a one-year life span and body length of 60–110 mm [3]. *P. fissuroides* inhabits warm, high-salinity tropical waters with sandy and muddy bottom sediments, occurring in deep waters from India eastward to the East China Sea [4]. The species is mainly harvested through drag operations, especially by beam shrimp trawling [3].

In China, its potential resource capacity was estimated to be 20–30 thousand tons [3]. *P. fissuroides* has been commercially exploited since the late 1980s in China, due to overexploitation of other shrimp species in coastal waters [3]. In Fujian, the annual production from shrimp trawling was estimated to be 4729–14,780 tons from 2005 to 2008, with an average value of 8810 tons [5]. Wang et al. (2014) and Ye et al. (2006) confirmed that the minimum body length and weight at first sexual maturity were 82 mm and 4.4 g, and 83 mm and 5.5 g, respectively [5,6].

In addition, shrimp species face threats from overfishing and climate change [7]. Heavy exploitation can lead to a decrease in catch per unit effort and individual sizes, while climate change can directly and indirectly influence the biomass, reproduction, and distribution patterns of marine organisms [8]. Climate change causes warming of the Earth’s oceans [9]. Invertebrates are more sensitive than fish to climate change [10]. Hadley Centre Sea Ice and Sea Surface Temperature (HadISST) observation showed that the largest warming trend has occurred in the East China Seas (including Bohai Sea, Yellow Sea, East China Sea) since the Industrial Revolution (1871 to 2014), with values up to 1.5 and 1.2 °C/century in boreal winter and summer [11]. The global climate models supported by the World Climate Research Program of Coupled Model Intercomparison Project Phase 6 were used to generate the projected climate scenarios under Shared Socio-Economic Pathways (SSPs) of low-(global radiative forcing of 2.6 W m^−2^), medium-(global radiative forcing of 4.5 W m^−2^), medium–high-(global radiative forcing of 7.0 W m^−2^), and high-end (8.5 W m^−2^) emissions, namely SSP126, SSP245, SSP370, and SSP585 [12]. In the East China Sea, Xu et al. (2025) found that *Metapenaeopsis provocatoria* received no great gains in habitat area under the four Shared Socioeconomic Pathway (SSP) scenarios (SSP1–2.6, SSP2–4.5, SSP3–7.0, and SSP5–8.5) in the 2040s and 2090s, and *Ovalipes punctatus* may migrate northward and offshore under these scenarios [13,14]. Marine organisms are also expected to shift their geographic distribution ranges and areas, thus affecting the distributions and catches of existing fisheries [15]. This poses a challenge for sustainable fishery management under heavy fishing pressure and climate change [15].

Currently, there is little knowledge on the seasonal–spatial distribution and migration patterns of *P. fissuroides*, including the responses of *P. fissuroides* to various climate scenarios, posing a barrier to sound fishery management in China. In this study, we aim to identify variations in the biomass (CPUE_w_), number (CPUE_n_), and average individual weight (AIW) across seasons and fishing grounds, in relation to environmental factors (such as sea surface temperature and salinity, sea bottom temperature and salinity), and the variation in habitat area change under climate scenarios (SSP1–2.6, SSP2–4.5, SSP3–7.0 and SSP5–8.5) in the 2040s and 2090s. The findings are essential for understanding changes in distribution patterns and fishery production over four decades, and finally providing management recommendations on this fishery.

## 2. Materials and Methods

**Figure 1:**
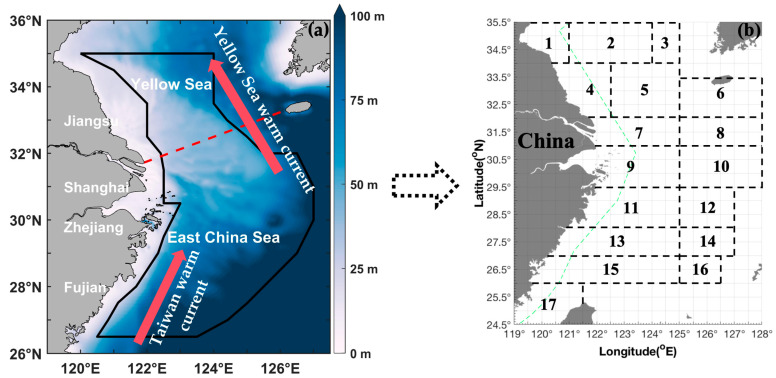
(**a**) Map of the study area (24.50–35.50° N, 119.00–128.00° E); study area is denoted by a red dotted line in the East China Sea region and includes the Southern Yellow Sea and East China Sea. The red arrows represent Yellow Sea warm Current and Taiwan warm Current. (**b**) Black boxes and numbers represent the following fishing grounds: (1) Haizhou Bay, (2) Lianqingshi, (3) Liandong, (4) Lvsi, (5) Dasha, (6) Shawai, (7) Yangtze River mouth, (8) Jiangwai, (9) Zhoushan, (10) Zhouwai, (11) Yushan, (12) Yuwai, (13) Wentai, (14) Wenwai, (15) Mindong, (16) Minwai, and (17) Minzhong. Green dashed line indicates the motor trawl prohibition lines.

Independent scientific bottom trawling surveys were undertaken in 2018 and 2019 at the East China Sea region (including the Southern Yellow Sea and the East China Sea) from spring to winter (Figure 1). A trawl net was used with headline of 72.24 m, height of 10–15 m, a cod end mesh size of 20 mm, and groundline of 82.44 m. The samples were transported to the laboratory for species identification. *Parapenaeus fissuroides* can be identified: rostrum extending beyond eyes with 6–7 upper teeth and without lower teeth, carapace with longitudinal and vertical sutures, telson with a pair of large subapical fixed lateral spines and without movable lateral spines, petasma in males symmetrical with subdistolateral bifurcate lobes, median part of thelycum in females bearing a pair of longitudinal swellings [16,17]. The total number of *P. fissuroides* samples in each survey station was counted and weighed to the nearest 0.10 g wet weight, and the catch density of *P. fissuroides* was calculated as biomass density per unit of sampling time (CPUE_w_; g·h^−1^) and as individual numerical density per unit of sampling time (CPUE_n_; ind·h^−1^). AIW was calculated as the CPUE_w_ divided by the CPUE_n_ at each station. The environmental variables were measured at each station using an SBE-19 profiler (Sea-Bird Scientific, Bellevue, WA, USA). Sea surface temperature (SST) and sea surface salinity (SSS) were measured 3 m below the surface, whereas sea bottom temperature (SBT) and sea bottom salinity (SBS) were measured 2 m above the sea bottom at depths of <50 m and at 2–4 m above the bottom at depths > 50 m.

In this study, we used the species distribution model (SDM) to describe and forecast the relationship between the environmental variables and species [18,19]. This study used “biomod2” package in the SDM ensemble platform (4.3–4) (https://biomodhub.github.io/biomod2/, accessed on 11 December 2025) in R software (version 3.4.6) [20]. The four Shared Socioeconomic Pathway (SSP) scenarios (SSP1–2.6, SSP2–4.5, SSP3–7.0, and SSP5–8.5) for 2040–2050 (the 2040s) and 2090–2100 (the 2090s) were used in this study [21]. The details and results of the survey procedure and SDM are detailed in Supplementary File S1.

## 3. Results

### 3.1. Seasonal Variations in Environmental Conditions

**Figure 2:**
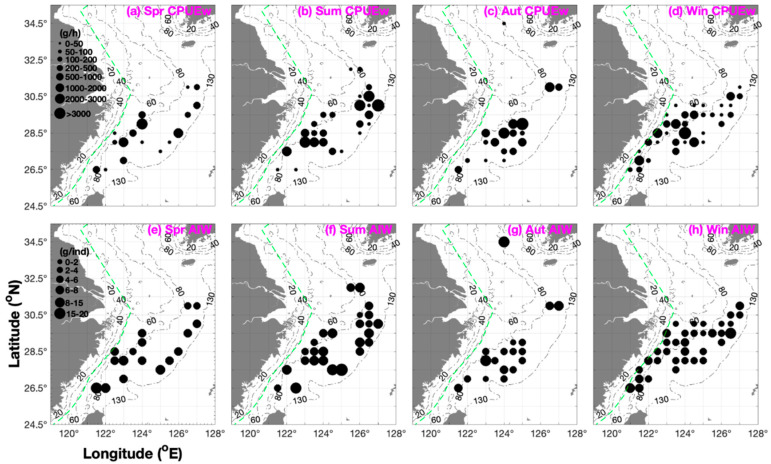
Seasonal distribution patterns of *Parapenaeus fissuroides* catch per unit effort by weight (CPUE_w_; g·h^−1^), depicted in black (grouped into 0–50, 50–100, 100–200, 200–500, 500–1000, 1000–2000, 2000–3000, and >3000 g·h^−1^), and the average individual weight (AIW; g·ind^−1^) data are shown in black (grouped into 0–2, 2–4, 4–6, 6–8, 8–15, and 15–20 g·ind^−1^). (**a**–**d**) CPUE_w_ in (**a**) spring, (**b**) summer, (**c**) autumn, and (**d**) winter. (**e**–**h**) AIW in (**e**) spring, (**f**) summer, (**g**) autumn, and (**h**) winter. Green dashed line indicates the motor trawl prohibition lines.

The depth range of *P. fissuroides* was 50–120 m throughout the year (Table 1). When CPUE_w_ > 1000 g·h^−1^, *P. fissuroides* was concentrated at a depth of 70–110 m in spring and summer, 80–90 m in autumn, and 60–100 m in winter (Figure 2). The fishing ground rankings were Yuwai (>100 m) > Zhouwai (90–100 m) > Wentai and Mindong (70–110 m) > Yushan (60–80 m) in spring; Yuwai (100–120 m) > Wentai, Mindong, and Zhouwai (70–100 m) > Jiangwai and Yushan (60–85 m) in summer; and Mindong (80–120 m) > Zhouwai (80–100 m) > Yushan and Wentai (70–100 m) in autumn (Table 2).

**Figure 3:**
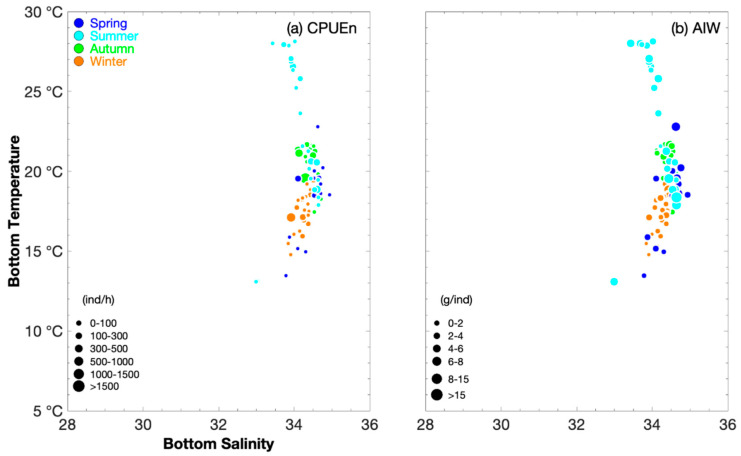
Relationship between bottom salinity and bottom temperature (°C) for catch per unit effort by number (CPUE_n_), classified by group (0–100, 100–300, 300–500, 500–1000, 1000–1500, and >1500 ind·h^−1^), and average individual weight (AIW), classified by group (0–2, 2–4, 4–6, 6–8, 8–15, and >15 g·ind^−1^), for *Parapenaeus fissuroides*. Data for spring, summer, autumn, and winter are denoted by blue, light blue, green, and brown circles, respectively. (**a**) Sea bottom temperature vs. sea bottom salinity for CPUE_n_, and (**b**) sea bottom temperature vs. sea bottom salinity for AIW.

Our findings showed that the lower limit value of SBT in summer was less than that in autumn (Table 1). The greatest *P. fissuroides* abundance occurred at SBTs of 18–20 °C in spring, 18–21 °C in summer, 19–22 °C in autumn, and 17–19 °C in winter (Figure 2). When AIW > 5 g·ind^−1^, SBT values were 18–23 °C in spring, 13–28 °C in summer, 18–22 °C in autumn, and 18–19 °C in winter (Figure 3). The fishing ground rankings in SST were Mindong (24–25 °C) > Wentai (21–23 °C) > Yushan (19–21 °C) > Yuwai (17–20 °C) > Zhouwai (16–17 °C) in spring; Zhouwai (28–30 °C) > Jiangwai, Yushan, and Yuwai (28–29 °C) > Wentai (26–29 °C) > Mindong (26 °C) in summer; and Mindong (23–25 °C) > Wentai and Yushan (22–24 °C) > Zhouwai (23 °C) in autumn (Table 2). The fishing ground rankings in SBT were Mindong (18–23 °C) > Yushan and Wentai (18–20 °C) > Yuwai (16–19 °C) > Zhouwai (13–15 °C) in spring; Yushan (21–28 °C) > Wentai, Mindong, and Yuwai (18–28 °C) > Zhouwai (19–22 °C) > Jiangwai (13 °C) in summer; and Yushan and Wentai (20–22 °C) > Zhouwai (19–21 °C) > Mindong (17–21 °C) in autumn (Table 2). In winter, the fishing ground rankings in SST and SBT were Mindong (18–19 °C) > Yuwai (17–19 °C) > Zhouwai and Wentai (16–19 °C) > Zhoushan and Yushan (15–17 °C) (Table 2).

The greatest *P. fissuroides* abundance (and when AIW > 5 g·ind^−1^) occurred at SBS values of 34–35 across all seasons (Figure 3), indicating the influence of the high-salinity warm current. The fishing ground rankings in SSS were Mindong (34–35) > Wentai (33–34) > Zhouwai (32–34) > Yushan and Yuwai (31–34) in spring; Yuwai, Wentai, and Mindong (33–34) > Yushan (32–34) > Zhouwai (31–33) > Jiangwai (28–29) in summer; Zhouwai and Wentai (34) > Yushan and Mindong (33.5–34.5) in autumn; and the same values were observed among Zhoushan, Zhouwai, Yushan, and Mindong (33–35), and between Yuwai and Wentai (34–35) in winter (Table 2). The fishing ground rankings in SBS were Yushan, Wentai, and Mindong (34–35) > Zhouwai and Yuwai (34–34.5) in spring; and Zhouwai and Mindong (34–35) > Yushan, Yuwai, and Wentai (33.5–34.5) > Jiangwai (33) in summer; the same values were found in the fishing grounds in autumn and winter (34–35) (Table 2).

### 3.2. Seasonal Variations in CPUE_w_, CPUE_n_, and AIW

Total CPUE_w_ and CPUE_n_ were 6610.1 g·h^−1^ and 1377.5 ind·h^−1^ in spring; 21,426.1 g·h^−1^ and 3481.8 ind·h^−1^ in summer; 22,892.2 g·h^−1^ and 7920.7 ind·h^−1^ in autumn; and 15,566.2 g·h^−1^ and 4438.1 ind·h^−1^ in winter (Figure 2). The annual mean CPUE_w_ and CPUE_n_ values were 16,623.65 g·h^−1^ and 4304.525 ind·h^−1^ (Figure 2). The seasonal order of total CPUE_n_ was autumn > winter > summer > spring (Table 3). In this study, the mean and upper limit values of CPUE_w_ and CPUE_n_ ranked ‘autumn > summer and winter > spring’, and the mean AIW ranked ‘summer > spring > autumn > winter’ (Table 3).

The mean CPUE_w_ and CPUE_n_ rankings for the fishing grounds in spring were Yushan and Yuwai (~70%) > Zhouwai and Wentai and Mindong (~30%), but AIW ranking was Mindong > Wentai > Zhouwai and Yushan and Yuwai (Table 2). In the summer, mean CPUE_w_ and CPUE_n_ rankings were Zhouwai (>0%) > Wentai (~25%) > Yushan and Yuwai (~10%) > Jiangwai and Mindong (~1%), indicating northward spawning migration from Yushan to Zhouwai during this period (Table 2). The AIW ranking was Wentai > Jiangwai and Mindong > Yuwai > Zhouwai and Yushan, indicating the presence of juveniles in Zhouwai and Yushan, with some juveniles migrating from Zhouwai to the Yuwai fishing grounds (Table 2). In autumn, the mean CPUE_w_ and CPUE_n_ rankings were Yushan (~70–80%) > Zhouwai and Wentai > Mindong, indicating that the Yushan fishing ground in the middle Zhejiang was a key nursery and feeding ground for *P. fissuroides*, with an AIW ranking of Zhouwai and Wentai > Yushan and Mindong (Table 2). In winter, the mean CPUE_w_ and CPUE_n_ rankings were Yushan (50%) > Wentai and Mindong (30%) > Zhouwai (10%) > Zhoushan and Yuwai (<5%), indicating potential overwintering ground in Yushan, Wentai, and Mindong fishing grounds, and the mean AIW ranking was Mindong > Yuwai and Wentai > Zhouwai and Yushan > Zhoushan (Table 2).

The longitudinal ranking for mean CPUE_w_ and CPUE_n_ in spring was 124° E > 126–127° E and 121–123.5° E > 125–125.5° E (Figure 2). In summer, the mean CPUE_w_ and CPUE_n_ rankings were 126–127° E > 121–124.5° E > 125–125.5° E (Figure 2). In autumn, the mean CPUE_w_ and CPUE_n_ rankings were ‘125° E > 123–124.5° E’, and ‘126.5–127° E > 121.5–122° E’ (Figure 2). In winter, the mean CPUE_w_ and CPUE_n_ rankings were 121–124.5° E > 125–127° E (Figure 2). Our findings suggested they move from the east of 125° E to the longitudinal line of 124° E (Figure 2 and Table 2).

In addition to AIW, the longitudinal ranking was 121.5–124° E > 125–127° E in spring; 124–127° E > 121.5–123.5° E in summer; 124–127° E > 121.5–123.5° E in autumn; and 121–121.5° E and 127° E > 122–126.5° E in winter (Figure 2).

### 3.3. Habitat Area Change Under Various Climate Scenarios

In this study, the ranking for suitable habitat area losses under the different climate scenarios was as follows: SSP585–2100 (about 60%) > SSP370–2100 (about 35%) > SSP245–2100 (about 20%) > SSP126–2100 and SSP370–2050 (about 10%) > SSP126–2050, SSP245–2050, and SSP585–2050 (about 0%). These results indicate area losses under different climate scenarios in the 2090s and almost no losses under climate scenarios (SSP126, SSP245, and SSP585) in the 2040s (Table 4). No more than 5% suitable habitat area was gained under climate scenarios in the 2040s and 2090s (Table 4). Generally, climate change adversely affects the habitat range of *P. fissuroides*.

## 4. Discussion

### 4.1. Spatial Distribution Pattern and Migration

*Parapenaeus fissuroides* is mainly distributed east of 124° E in the Central and Southern East China Sea at depths > 60 m. Song et al. (2002) reported the highest biomass around 100 m depth in spring, peaking at 2420 g·h^−1^, and they suggested that the distribution range was influenced by the Taiwan Warm Current [3]. Wang et al. (2014) identified the biomass value in the depth ranking of 80–100 m > 60–80 m > 40–60 m > less than 40 m [5]. Likewise, Song et al. (2002) suggested that *P. fissuroides* was mainly distributed south of the Yushan fishing ground (26.00–30.00° N, east of 60 m), with a dense concentration distribution in the offshore waters of Central and Southern Zhejiang, but a low density south of 26.50° N [3]. In addition, one existing record in this study was observed in the Southern Yellow Sea in autumn (Figure 2), indicating that Yellow Sea Warm Current may support a very small population of *P. fissuroides* in the water areas between Korea and China.

**Figure 4:**
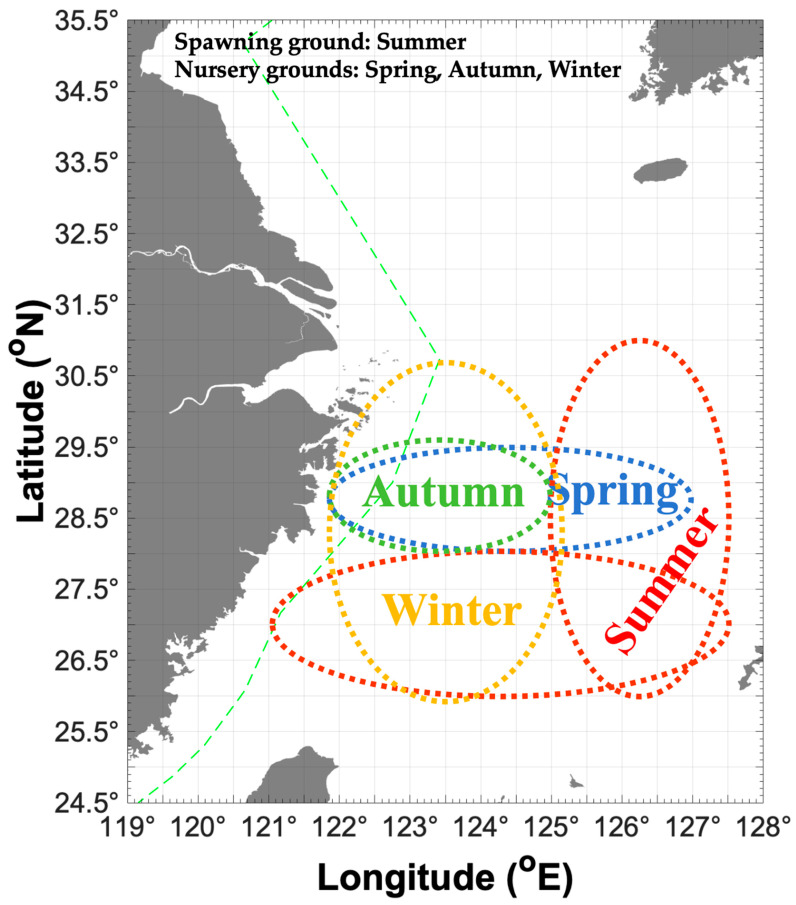
Map of possible distribution patterns for *Parapenaeus fissuroides* across seasons in China, showing the spawning ground in summer (indicated by red dotted ellipse) and nursery grounds in spring (indicated by blue dotted ellipse), autumn (indicated by green dotted ellipse), and winter (indicated by yellow dotted ellipse). Green dashed line indicates the motor trawl prohibition lines.

In this study, we addressed the assumption about the potential migration route of *P. fissuroides.* We assume that, in spring, the recruitment cohorts from the previous year are concentrated in the Yushan and Yuwai fishing grounds for the upcoming spawning season (Figure 4). In summer, the major parent cohorts release offspring in high-temperature and high-salinity water (SBT 18–21 °C and SBS 34–35) in the Zhouwai and Yuwai fishing grounds (Figure 4). Additionally, a portion of parent groups is concentrated in the Wentai and Mindong fishing grounds at SBT 18–27 °C and SBS 34–35. In autumn, most of the newborn offspring migrate to the Yushan fishing ground for nursery. During winter, most recruitment cohorts concentrate in Yushan, Wentai, and Mindong fishing grounds for accelerated growth during this season (Figure 4). Song et al. (2002) found the most suitable SBTs for *P. fissuroides* were 17–24 °C in summer and 14–18 °C in winter in the East China Sea [3].

### 4.2. Fishery Management Strategies and Response to Climate Change

Conservation and protection of parent and recruitment cohorts are vitally important to sustainable development of *P. fissuroides* fisheries, e.g., setting seasonal area closure in the offshore waters of Central and Southern Zhejiang in China. Regarding the breeding period, Song et al. (2002) suggested the breeding period is from July to October, peaking in August in China [3]; in Kagoshima Bay, Kyushu, Japan, the spawning season of *P. fissuroides* was from July to February, with a peak in October–November [22]. In China’s water area, the newborn offspring occurred in August, and growth was accelerated in spring of the following year, with maximum body length in August to September [3]. Reportedly, the gonadal maturity of *P. fissuroides* attained stage III in March to June, and stages IV and V in July to October, with a measured 40.9% individuals with stage V in August [3]. The overwintering cohort, with body length ranging from 50 to 75 mm, began rapid growth from April, becoming the dominant group, with body lengths of 50 to 85 mm in March to May and 65 to 105 mm body lengths in June to August, with maximum values of the female 80–110 mm, and the male 70–90 mm, in August to September [3].

Newborn individuals, with body lengths of 35–55 mm, were observed in August, attaining lengths of 55–75 mm in December, and two generations coexisted from August to December [3]. After October, the adult cohort number gradually decreased, and the newborn individual number gradually increased [3]. In the Northeast Fujian Sea, the cohort in spring primarily consisted of small patches with a low population aggregation; in summer, the population aggregation was the highest; in autumn, the cohort primarily consisted of a small number of large patches; and, in winter, the population aggregation was the lowest [4]. Ye et al. (2006) and Song et al. (2002) found the seasonal biomass order of spring > other seasons [3,6]; Cai et al. (2017) found the biomass ranking of summer > autumn > spring and winter [23]. In the Mindongbei Sea water area, Wang et al. (2014) obtained a mean CPUE_w_ value of 2300 g·h^−1^ during 1998–1999 and 1600 g·h^−1^ during 2008–2009, with the seasonal biomass and number order following ‘August (2821.6 g·h^−1^) > November (1742 g·h^−1^) > February (1423.3 g·h^−1^) > May (455.8 g·h^−1^)’ and ‘November (413.8 ind·h^−1^) > August (406.4 ind·h^−1^) > February (305.2 ind·h^−1^) > May (106.9 ind·h^−1^)’ [5].

In China’s exclusive economic zones, fishery operations are mainly multi-species, with ~90% of the total marine catch from non-selective trawlers, purse seines, gill nets, and set nets [24]. Given the diversity of shrimp species, ecological niches, and geographic ranges, breeding periods and peak offspring occurrence vary substantially [25]. For *P. fissuroides*, the peak recruitment period was from September to November, and juveniles prefer depths > 50–60 m. In this study, we address a closed season and distribution range (including Yushan, Yuwai, Zhouwai, and Mindong fishing grounds) during August to November for *P. fissuroides* to protect juveniles and females bearing eggs, which would help the recovery of the recruitment and spawning stocks to achieve the maximum sustainable yield for economic and biological goals. In addition, zooplankton number and biomass are major factors affecting population aggregation, so it is important to perform continuous seasonal biological monitoring from Zhoushan to Mindong fishing grounds.

Additionally, warmer seawater might reduce the size at maturity and cause mature individuals to spawn earlier in marine crustaceans [26], causing a failure or decrease in recruitment. Habitat area range loss and shifts in distribution of the recruitment cohorts may reduce the catch of fishery species and ultimately influence local fishing activities [27]. Given the adverse impacts of climate change on *P. fissuroides*, improved conservation and management practices are necessary to mitigate the consequences projected under future climate scenarios in the study area. Meanwhile, it is also necessary to report the current resource status of *P. fissuroides* and possible threats and challenges from climate change to multinational climate change organizations supported by governments to appeal for immediate changes in marine policies to avoid the regional resource collapse of *P. fissuroides*.

## 5. Conclusions

*Parapenaeus fissuroides* is a tropical and temperate regional shrimp species with high-water-temperature (suitable SBT > 18 °C) and high-salinity (suitable SBS > 34) habitat in East Asia. In China, the warm currents, including the Yellow Sea Warm Current and Taiwan Warm Current, which are branches of the Kuroshio current assumed to have important roles on the species’ spatial distribution pattern and migration. In addition, global warming asserted the most serious negative impacts on the East China Seas, with a possible 1.2–1.5 °C SST increase, so it is necessary to monitor long-term resource status and spatial distribution patterns of marine organisms, including *P. fissuroides*, in the study area. We suggested more seasonal closure actions in the interest of protecting the recruitment of *P. fissuroides* in Zhejiang of China. Our findings are valuable for regional fishery management and biodiversity protection.

## Figures and Tables

**Table 1:** Seasonal data ranges for sea surface temperature (SST), sea bottom temperature (SBT), sea surface salinity (SSS), and sea bottom salinity (SBS), and depth from autumn 2018 to summer 2019.

Factor	Spring	Summer	Autumn	Winter
SST (°C)	15.8–24.8	26.1–29.6	21.7–24.8	14.7–19.6
SBT (°C)	13.5–22.8	13.1–28.1	17.5–21.7	14.8–19.5
SSS	31.7–34.5	28.3–34.1	33.5–34.4	33.7–34.4
SBS	33.8–34.9	33–34.6	34.1–34.7	33.8–34.6
Depth (m)	63–107	62–120	70–115	46–114

**Table 2:** Mean and total values for catch per unit effort by weight (CPUE_w_; g·h^−1^), percentage CPUE_w_, catch per unit effort by number (CPUE_n_; ind·h^−1^), percentage CPUE_n_, and average individual weight (AIW; g·ind^−1^) under different environmental conditions, i.e., sea surface temperature (SST), sea surface salinity (SSS), sea bottom temperature (SBT), sea bottom salinity (SBS), and depth in different fishing grounds (see Figure 1), according to season.

Fishing Ground	Mean Value					Total Value				Environmental Variable				
	B	B%	N	N%	AIW	B	B%	N	N%	SST	SSS	SBT	SBS	Depth
Spring														
Zhouwai	174	8.6%	45	10.6%	4	521	7.9%	136	9.9%	15.8–17.2	32.3–33.9	13.5–15.2	33.8–34.3	86–103
Yushan	693	34.2%	157	36.7%	5	2772	41.9%	627	45.5%	18.9–20.5	31.7–33.5	19.5–20	34.1–34.5	63–76
Yuwai	679	33.5%	148	34.5%	4	1357	20.5%	295	21.4%	16.7–20.1	31.7–33.9	15.9–18.5	33.9–34.5	101–107
Wentai	257	12.7%	43	10.1%	6	1287	19.5%	215	15.6%	20.8–22.9	33–34	18.6–20.2	34.7–34.8	72–104
Mindong	224	11.1%	35	8.1%	7	672	10.2%	104	7.6%	23.7–24.8	34.3–34.5	18.5–22.8	34.6–34.9	75–107
Summer														
Jiangwai	33	0.9%	5	0.8%	7	67	0.3%	9	0.3%	28.2–28.5	28.3–28.5	13.1	33	62–77
Zhouwai	2048	55.2%	319	53.1%	5	12,286	57.3%	1913	54.9%	28.2–29.6	31.1–32.6	18.9–21.6	34.2–34.6	73–97
Yushan	312	8.4%	70	11.7%	5	1873	8.7%	421	12.1%	27.7–28.9	31.9–33.7	21.3–28	33.4–34.4	69–84
Yuwai	288	7.8%	42	7%	6	1151	5.4%	167	4.8%	28.4–28.8	33.5–33.8	18.8–28.1	33.7–34.5	97–120
Wentai	997	26.9%	160	26.6%	9	5984	27.9%	961	27.6%	26.4–28.7	33.8–34	17.9–27.1	33.9–34.6	75–101
Mindong	33	0.9%	6	0.9%	7	66	0.3%	11	0.3%	26.1–26.3	33.7–34.1	19.5–26.3	34–34.4	75–104
Autumn														
Zhouwai	777	17.5%	155	10.6%	6	1554	6.8%	309	3.9%	22.7–23.1	34	18.6–21.1	34.5–34.6	84–100
Yushan	3129	70.5%	1141	78.5%	3	18,774	82%	6847	86.5%	21.7–23.4	33.7–34.3	19.6–21.7	34.1–34.4	70–100
Wentai	428	9.6%	134	9.2%	5	2141	9.4%	672	8.5%	23–23.9	34–34.2	19.8–21.6	34.5–34.6	76–100
Mindong	106	2.4%	23	1.6%	3	423	1.8%	92	1.2%	22.9–24.8	33.5–34.4	17.5–20.9	34.3–34.7	76–115
Winter														
Zhoushan	18	0.9%	12	2%	2	55	0.4%	36	0.8%	14.7–16.6	33.8–34.3	14.8–16.6	33.8–34.4	55–66
Zhouwai	158	7.6%	61	10.2%	3	791	5.1%	304	6.8%	16.3–19.2	33.9–34.3	16–19.2	34–34.3	81–100
Yushan	1119	53.6%	311	52.2%	3	10,074	64.7%	2798	63%	15–17.3	33.8–34.3	15.2–17.2	33.9–34.4	60–85
Yuwai	74	3.5%	27	4.6%	4	442	2.8%	165	3.7%	17.1–18.7	34.1–34.4	17.4–18.8	34.3–34.6	86–114
Wentai	334	16%	99	16.6%	4	2672	17.2%	791	17.8%	16.1–19.2	34.1–34.3	16.3–19.5	34.1–34.5	46–107
Mindong	383	18.4%	86	14.4%	5	1532	9.8%	344	7.8%	17.6–19.6	33.7–34.3	18–19.4	34.2–34.5	50–100

**Table 3:** Seasonal data for catch per unit effort by weight (CPUE_w_; g·h^−1^), number (CPUE_n_; ind·h^−1^), and average individual weight (AIW; g·ind^−1^) from autumn 2018 to summer 2019.

Factor	Spring	Summer	Autumn	Winter
Mean CPUE_w_ at collection stations	388.8	824.1	1346.6	444.7
Value range of CPUE_w_	5.4–2092.8	9–6863.4	1.5–13,478.3	3.3–6003.2
Mean CPUE_n_ at collection stations	81	133.9	465.9	126.8
Value range of CPUE_n_	1–464	1.1–920.7	1–4608	1–1408
Mean AIW	5.3	6.3	3.9	3.5
Value range of AIW	2.5–9.9	1.3–17.3	1.5–11.7	0.5–11.7

**Table 4:** Percentage habitat loss, gain, and overall habitat (gain minus loss) for *Parapenaeus fissuroides* under various climate scenarios (SSP126–2050, SSP126–2100, SSP245–2050, SSP245–2100, SSP370–2050, SSP370–2100, SSP585–2050, and SSP585–2100). SSP: Shared Socioeconomic Pathway.

Case	Loss%	Gain%	Gain% − Loss%
SSP126–2050	–0.75	2.97	2.22
SSP126–2100	–8.47	1.76	–6.71
SSP245–2050	–1.39	2.46	1.08
SSP245–2100	–17.34	1.19	–16.15
SSP370–2050	–8.12	1.83	–6.29
SSP370–2100	–34.68	1.19	–33.49
SSP585–2050	–1.5	3.45	1.96
SSP585–2100	–56.85	1.19	–55.67

## Data Availability

The original contributions presented in this study are included in the article. Further inquiries can be directed to the corresponding author(s).

## Supplementary Materials

The following supporting information can be downloaded at https://www.mdpi.com/article/10.3390/ani15243597/s1. File S1: More Information about materials and methods; Figure S1. Ratio values of TSS vs. ROC with x-direction and y-direction error bars, produced by the artificial neural network (ANN), classification tree analysis (CTA), flexible discriminant analysis (FDA), generalized additive model (GAM), generalized boosting model (GBM), generalized linear model (GLM), multiple adaptive regression splines (MARS), random forest (RF), surface range envelope (SRE), and extreme gradient boosting training (XGBOOST) methods; Figure S2. Calibration percentage (%) of TSS and ROC with the artificial neural network (ANN), classification tree analysis (CTA), flexible discriminant analysis (FDA), generalized additive model (GAM), generalized boosting model (GBM), generalized linear model (GLM), multiple adaptive regression splines (MARS), random forest (RF), surface range envelope (SRE), and extreme gradient boosting training (XGBOOST) method; Figure S3. Predicted spatial habitat distribution patterns of *Parapenaeus fissuroides* in the cases of (a) annual mean habitat, (b) SSP126 in 2050, (c) SSP126 in 2100, (d) SSP245 in 2050, (e) SSP245 in 2100, (f) SSP370 in 2050, (g) SSP370 in 2100, (h) SSP585 in 2050, and (i) SSP585 in 2100. Ref. [28] is cited in Supplementary Materials.
